# What Is the Contribution of Two Genetic Variants Regulating VEGF Levels to Type 2 Diabetes Risk and to Microvascular Complications?

**DOI:** 10.1371/journal.pone.0055921

**Published:** 2013-02-06

**Authors:** Amélie Bonnefond, Pierre-Jean Saulnier, Maria G. Stathopoulou, Niels Grarup, Ndeye Coumba Ndiaye, Ronan Roussel, Mohsen Azimi Nezhad, Aurélie Dechaume, Olivier Lantieri, Serge Hercberg, Torsten Lauritzen, Beverley Balkau, Julia S. El-Sayed Moustafa, Torben Hansen, Oluf Pedersen, Philippe Froguel, Guillaume Charpentier, Michel Marre, Samy Hadjadj, Sophie Visvikis-Siest

**Affiliations:** 1 “Cardiovascular Genetics” Research Unit, EA4373, University of Lorraine, Nancy, France; 2 Centre Hospitalier Universitaire de Poitiers, Department of Endocrinology and Diabetology, University of Poitiers, Poitiers, France; 3 Clinical investigation centre CIC0802, Poitiers, France; 4 Novo Nordisk Foundation Center for Basic Metabolic Research, Copenhagen, Denmark; 5 Faculty of Health Sciences, University of Copenhagen, Copenhagen, Denmark; 6 Steno Diabetes Center and Hagedorn Research Institute, Gentofte, Denmark; 7 Department of Endocrinology, Diabetology and Nutrition, Bichat-Claude Bernard University Hospital, Assistance Publique des Hôpitaux de Paris (AP-HP), Paris, France; 8 Institut National de la Santé et de la Recherche Médicale (Inserm) U695, Paris 7 University, Paris, France; 9 Centre National de la Recherche Scientifique (CNRS) UMR8199, Lille Pasteur Institute, Lille, France; 10 Lille Nord de France University, Lille, France; 11 Institut inter-régional pour la santé (IRSA), La Riche, France; 12 Inserm-U557, Institut National de la Recherche Agronomique (INRA) 1125 Unit, Conservatoire National des Arts et Métiers, Centre de Recherches en Nutrition Humaine, Paris 13 University, Bobigny, France; 13 Department of Public Health, Avicenne Hospital, AP-HP, Bobigny, France; 14 Department of General Practice, University of Aarhus, Aarhus, Denmark; 15 Inserm-U1018, Centre for Research in Epidemiology and Population Health, Villejuif, France; 16 Paris-Sud 11 University, Villejuif, France; 17 Department of Genomics of Common Disease, School of Public Health, Imperial College London, Hammersmith Hospital, London, United Kingdom; 18 Faculty of Health Sciences, University of Aarhus, Aarhus, Denmark; 19 Department of Endocrinology and Diabetology, Corbeil-Essonnes Hospital, Essonnes, France; 20 Inserm-U1082, Poitiers, France; Università di Roma “Sapienza”, Italy

## Abstract

Vascular endothelial growth factor (VEGF) is a key chemokine involved in tissue growth and organ repair processes, particularly angiogenesis. Elevated circulating VEGF levels are believed to play a role in type 2 diabetes (T2D) microvascular complications, especially diabetic retinopathy. Recently, a genome-wide association study identified two common single nucleotide polymorphisms (SNPs; rs6921438 and rs10738760) explaining nearly half of the variance in circulating VEGF levels. Considering the putative contribution of VEGF to T2D and its complications, we aimed to assess the effect of these VEGF-related SNPs on the risk of T2D, nephropathy and retinopathy, as well as on variation in related traits.

SNPs were genotyped in several case-control studies: French and Danish T2D studies (*N_cases_* = 6,920-*N_controls_* = 3,875 and *N_cases_* = 3,561-*N_controls_* = 2,623; respectively), two French studies one for diabetic nephropathy (*N_cases_* = 1,242-*N_controls_* = 860) and the other for diabetic retinopathy (*N_cases_* = 1,336-*N_controls_* = 1,231). The effects of each SNP on quantitative traits were analyzed in a French general population-based cohort (*N* = 4,760) and two French T2D studies (*N* = 3,480). SNP associations were assessed using logistic or linear regressions.

In the French population, we found an association between the G-allele of rs6921438, shown to increase circulating VEGF levels, and increased T2D risk (OR = 1.15; *P* = 3.7×10^−5^). Furthermore, the same allele was associated with higher glycated hemoglobin levels (β = 0.02%; *P* = 9.2×10^−3^). However, these findings were not confirmed in the Danes. Conversely, the SNP rs10738760 was not associated with T2D in the French or Danish populations. Despite having adequate statistical power, we did not find any significant effects of rs6921438 or rs10738760 on diabetic microvascular complications or the variation in related traits in T2D patients.

In spite of their impact on the variance in circulating VEGF, we did not find any association between SNPs rs6921438 and rs10738760, and the risk of T2D, diabetic nephropathy or retinopathy. The link between VEGF and T2D and its complications might be indirect and more complex than expected.

## Introduction

Vascular endothelial growth factor A (VEGFA – also known as VEGF) is a key chemokine involved in tissue growth and organ repair processes. Primarily, the role of VEGF has been intensely investigated in the regulation of angiogenesis, namely the growth of novel vascular endothelial cells derived from arteries, veins or lymphatics, which is essential for organ development and tissue repair, but also for tumor growth [1], [2]. VEGF mediates angiogenesis by increasing vascular permeability to water and proteins [3]. However, the excessive vascular permeability during pathological angiogenesis can contribute to both macro- and microvascular diseases [3]. In this regard, higher circulating VEGF levels have been detected in the serum of some patients presenting with various types of cancer [4], with cardiovascular diseases [5], [6], [7] or with diabetic microvascular complications [8]. Higher levels of serum VEGF play a central role in the development of diabetic retinopathy, and intravitreal anti-VEGF drugs are currently widely used in patients presenting with proliferative diabetic retinopathy or diabetic macular edema [8].

The heritability of circulating VEGF levels is very high, estimated at between 60 and 80% [9], [10], [11]. A recent genome-wide association study (GWAS) reported several common single nucleotide polymorphisms (SNPs) that were significantly associated with serum VEGF levels [12]. In particular, two SNPs explained a very large proportion of the heritability of circulating VEGF levels in the Framingham study: rs6921438 and rs10738760, explaining 41.2% and 5.0% of the VEGF variance, respectively [12]. SNP rs6921438 is located on chromosome 6p21.1, at 171 kb downstream of *VEGFA*, and close to the *C6orf223* gene (which encodes an uncharacterized protein); and SNP rs10738760 is located on chromosome 9p24.2, between the *VLDLR* and *KCNV2* genes (that encode the very low density lipoprotein receptor and the potassium voltage-gated channel subfamily V, member 2, respectively).

We postulated that if increased circulating VEGF is genuinely a causative risk factor for type 2 diabetes (T2D) and/or its microvascular complications, then the SNPs rs6921438 and rs10738760, which explain nearly half the variance in circulating VEGF, would also contribute to the genetic risk of T2D and its microvascular complications. Therefore, we aimed to assess the association of both these VEGF-related SNPs (rs6921438 and rs10738760) with the risk of T2D, diabetic nephropathy and retinopathy, and with variation in related metabolic traits, in European populations.

## Materials and Methods

### Study participants

Clinical characteristics and data available for the studied populations are reported in **Table S1**.

Genotyping of SNPs rs6921438 and rs10738760 was conducted in several study samples:

- *French T2D case-control study*. We analysed 6,920 unrelated French individuals with T2D ascertained from the French T2D family study collected by the CNRS-UMR8090 unit, from the Endocrinology-Diabetology Department of the Corbeil-Essonnes Hospital [13], from the Diabhycar/Diab2-Néphrogène study [14], from the D.E.S.I.R. cohort which is a longitudinal French general population sample [15], and from the SU.VI.MAX study, which is fully described elsewhere [16]. We used 3,875 unrelated normoglycemic adults (age at exam ≥45 years), ascertained from the D.E.S.I.R. and the SU.VI.MAX studies, as controls.
- *Danish T2D case-control study*. We analysed a total of 3,561 Danish patients with T2D recruited by the Steno Diabetes center [17], as well as the Inter99 [18] and ADDITION studies [19]. We used 2,623 unrelated normoglycemic adults (age at exam ≥45 years), recruited by the Steno Diabetes center and the Inter99 study, as controls.
- *D.E.S.I.R.* The Data from the Epidemiological Study on the Insulin Resistance Syndrome (D.E.S.I.R.) cohort is a longitudinal French general population sample, fully described elsewhere [15]. We assessed the association of SNPs rs6921438 and rs10738760 with variation in quantitative metabolic traits (fasting glucose, fasting insulin, glycated haemoglobin [A1c], homeostasis model of pancreatic beta-cell function [HOMA-B] and homeostasis model of insulin resistance [HOMA-IR]) in 4,760 non-diabetic D.E.S.I.R. participants.
- *Corbeil study*. We assessed the association of SNPs rs6921438 and rs10738760 with variation in quantitative traits related to T2D microvascular complications (estimated glomerular filtration rate [eGFR], urinary albumin/creatinine ratio [ACR]) in 1,970 participants with T2D from Corbeil [13].
- *Diab2-Néphrogène (D2NG) study*. We assessed the association of SNPs rs6921438 and rs10738760 with variation in quantitative traits related to T2D microvascular complications (eGFR and ACR) in 1,510 participants with T2D from the D2NG study [14].
- *Case-control study for diabetic nephropathy and retinopathy*. Participants with T2D from Corbeil or the D2NG study were included in this analysis.

In the Corbeil study, we defined patients with a stage of kidney disease higher than 2 as cases (see details below) in the analyses for association with diabetic nephropathy (*N_Corbeil_* = 689). Normoalbuminuric controls were required to have a duration of diabetes ≥10 years (*N_Corbeil_* = 570). For the analysis of retinopathy, 506 T2D individuals from Corbeil were included as cases, while controls (*N_Corbeil_* = 591) were required to have a duration of diabetes ≥10 years and no signs of retinopathy. In cases, retinopathy was staged as background, severe non-proliferative or proliferative; and macular edema was staged as present/absent according to fundoscopy by a trained ophthalmologist, and a retinal angiography or an optical coherence tomography (OCT) when clinically indicated.

In the D2NG study, diabetic retinopathy was considered for all participants with a similar ophthalmologist-based classification (cases: *N_D2NG_* = 838; controls: *N_D2NG_* = 639). All patients were considered for their retinopathy stage. With regards to renal status, cases were identified as patients presenting with different stages of renal involvement (see details below) and with retinopathy (to ensure the specificity of diabetic nephropathy) (*N_D2NG_* = 551). Controls were normoalbuminuric and normal renal function patients with a known T2D duration ≥10 years (*N_D2NG_* = 287). Furthermore, any subjects taking angiotensin-converting-enzyme (ACE) inhibitors or angiotensin II receptor blockers (ARBs) were excluded from the control sample. This phenotype was not available in the Corbeil study, which constitutes a limitation of this study as ACE inhibitors or ARBs intake can influence the ACR and thus lead to a possible overestimation of the number of controls.

Glycemic status was defined according to 1997 American Diabetes Association criteria [20]: normal glucose, defined as fasting plasma glucose <6.1 mmol/l without hypoglycemic treatment; and T2D, defined as fasting plasma glucose ≥7.0 mmol/l and/or treatment with hypoglycemic agents.

Clinical data relating to nephropathy were defined as follows: i/Stage 2: microalbuminuria defined as ACR ≥30 mg/g [3.5 mg/mmol] and <300 mg/g [35 mg/mmol] (at three consecutive measurements); ii/Stage 3: macroalbuminuria defined as ACR ≥300 mg/g [35 mg/mmol] (at three consecutive measurements); and iii/Stages ≥4: eGFR <30 ml/min/1.73 m^2^, or patients undergoing dialysis or having received a kidney transplant.

All studies were approved by the local ethical committees (from France: CPP [Comité de Protection des Personnes] of Lille, Kremlin-Bicêtre, Corbeil-Essonnes, Poitiers and Paris; and from Denmark [Copenhagen]: ClinicalTrials.gov-Identifier #NCT00289237) as being in accordance with the Declaration of Helsinki. All study participants also gave written informed consent.

### Genotyping

All DNA samples used for the present study were extracted from blood. Genotyping of SNPs rs6921438 and rs10738760 was performed using TaqMan assays according to the manufacturer's instructions (Applied Biosystems; AB assay IDs C-11542106-10 and C-11257266-10, respectively). A genotyping success rate of at least 97% and no deviation from Hardy-Weinberg equilibrium (*P*>0.05) were observed in all the study populations. Furthermore, a total of 182 samples were also assessed by Sanger sequencing, and a concordance of more than 99% was observed for both SNPs.

### Indices calculation

The eGFR was calculated using the modification of diet in renal disease (MDRD) equation as follows:

eGFR = 175×(creatinine/88.4)^−1.154^×age^−0.203^×(0.742 if female), where creatinine is in µmol/l [21].

The HOMA-B was calculated as:

HOMA-B = (20×fasting serum insulin)/(fasting plasma glucose−3.5), where fasting serum insulin is in mU/l and fasting plasma glucose is in mmol/l [22].

The HOMA-IR was calculated as:

HOMA-IR = (fasting plasma glucose×fasting serum insulin)/22.5, where fasting plasma glucose is in mmol/l and fasting serum insulin is in pmol/l [22].

### Statistical analyses

We analyzed the effect of SNPs rs6921438 and rs10738760 on quantitative traits (fasting plasma glucose, fasting serum insulin, HOMA-B, HOMA-IR and A1c) using linear regression models under an additive model, adjusted for age and gender, or for age and duration of T2D (eGFR and ACR). Data for fasting serum insulin, HOMA-B, HOMA-IR and ACR were logarithmically transformed before statistical analysis.

The effect of SNPs rs6921438 and rs10738760 on risk of T2D, retinopathy or nephropathy was assessed using a logistic regression model adjusted for age and gender (T2D) or for T2D duration and gender (retinopathy and nephropathy).

The combined analyses were performed using a weighted inverse normal method via the function “metagen”, with a fixed effect, in the “META” R package. No heterogeneity was observed (*P*>0.1).

All statistical analyses were performed using SPSS (version 14.0 for Windows), except combined analyses and statistical power calculation which were performed using R (version 2.15.1 for Windows) and Quanto, respectively.

## Results

### Effect of SNPs rs6921438 and rs10738760 on the risk of T2D and variation in related metabolic traits

We firstly assessed the association between both SNPs rs6921438 and rs10738760, and T2D, in a large T2D French case-control study including 6,920 T2D patients and 3,875 normoglycemic controls.

Using a logistic regression adjusted for age and gender (under an additive model), we identified a significant association between the G-allele of rs6921438 (increasing circulating VEGF levels [12]) and increased T2D risk (odds ratio [95% confidence interval]: OR = 1.15 [1.07;1.22]; *P* = 3.74×10^−5^; **Table 1**). Of note, a meta-analysis of GWAS reported a significant association between a SNP located close to *VEGFA* and waist-hip ratio [23]. In order to assess whether the significant association between rs6921438 and T2D risk was driven by central obesity, the logistic regression was also adjusted for age, gender and BMI. We observed the same magnitude of association between rs6921438 and T2D risk after correction for BMI (OR = 1.17 [1.08;1.26]; *P* = 7.25×10^−5^; data not shown).

**Table 1:** Association of SNPs rs6921438 and rs10738760 with T2D risk in two European case-control studies.

SNPs	Studies	X frequency (%)	*N*	YY (%)	YX (%)	XX (%)	OR (95% CI)*	*P*
**rs6921438** (G-allele = X A-allele = Y)	**French controls**	52.5	3,745	861 (23.0)	1,834 (49.0)	1,050 (28.0)	-	-
	**French cases**	55.6	6,908	1,367 (19.8)	3,402 (49.2)	2,139 (31.0)	**1.15 (1.07;1.22)**	**3.7×10^−5^**
	**Danish controls**	50.8	2,600	590 (22.7)	1,324 (50.9)	686 (26.4)	-	-
	**Danish cases**	51.8	3,524	857 (24.3)	1,753 (49.7)	914 (25.9)	1.02 (0.94;1.10)	0.66
	**Combined analysis**	-	-	-	-	-	-	0.28
**rs10738760** (A-allele = X G-allele = Y)	**French controls**	50.4	3,756	916 (24.4)	1,896 (50.5)	944 (25.1)	-	-
	**French cases**	50.6	6,914	1,733 (25.1)	3,362 (48.6)	1,819 (26.3)	0.98 (0.91;1.06)	0.63
	**Danish controls**	51.2	2,592	611 (23.6)	1,308 (50.5)	673 (26.0)	-	-
	**Danish Cases**	50.9	3,512	827 (23.5)	1,796 (51.1)	889 (25.3)	1.04 (0.96;1.12)	0.40
	**Combined analysis**	-	-	-	-	-	-	0.93

* OR from additive logistic regression models adjusted for age and gender.

***T2D***, type 2 diabetes; ***OR***, odds ratio; ***CI***, confidence interval; ***P***, P-value.

Furthermore, the same allele was associated with higher glycated haemoglobin (A1c) levels in 4,600 nondiabetic French participants from the D.E.S.I.R. study (effect size (standard error): β = 0.020 (0.007) %_A1c_; *P* = 9.2×10^−5^; **Table 2**). However, no association with fasting glucose, fasting insulin, HOMA-B or HOMA-IR was observed (**Table 2**).

**Table 2:** Effect of SNPs rs6921438 and rs10738760 on variation in quantitative metabolic traits in nondiabetic participants from D.E.S.I.R.

			Mean/median data level by genotype				
SNPs	Metabolic traits	*N*	YY	YX	XX	β (SE)*	*P*
**rs6921438** (G-allele = X A-allele = Y)	**Fasting glucose (mmol/l)**	4,600	5.29±0.55	5.27±0.53	5.30±0.54	0.012 (0.010)	0.24
	**Fasting insulin (pmol/l)**	4,600	39.5 (29.4;53.9)	39.2 (28.4;56.5)	39.7 (28.7;56.1)	0.003 (0.011)	0.81
	**HOMA-B**	4,600	67.2 (51.0;92.9)	69.1 (48.4;96.2)	67.3 (48.2;93.9)	−0.009 (0.011)	0.38
	**HOMA-IR**	4,600	9.2 (6.5;13.2)	9.1 (6.4;13.6)	9.3 (6.5;13.6)	0.005 (0.012)	0.66
	**A1c (%)**	4,600	5.41±0.41	5.42±0.39	5.45±0.40	**0.020 (0.007)**	**9.2×10^−3^**
**rs10738760** (A-allele = X G-allele = Y)	**Fasting glucose (mmol/l)**	4,602	5.28±0.53	5.28±0.54	5.29±0.54	0.009 (0.010)	0.39
	**Fasting insulin (pmol/l)**	4,602	38.5 (28.6;55.2)	38.9 (28.5;55.7)	41.4 (29.7;57.2)	0.018 (0.009)	0.053
	**HOMA-B**	4,602	66.9 (48.4;94.5)	67.9 (48.1;94.3)	70.0 (51.8;97.5)	0.017 (0.011)	0.11
	**HOMA-IR**	4,602	8.9 (6.5;13.2)	9.1 (6.4;13.6)	9.8 (6.6;13.8)	0.020 (0.010)	0.051
	**A1c (%)**	4,602	5.43±0.39	5.43±0.40	5.43±0.40	0.006 (0.008)	0.47

* Per X-allele effect size: coefficient β from additive linear regression models adjusted for age and gender.

Data are presented as mean ± standard deviation or median (interquartile range). Data for fasting serum insulin, HOMA-B, and HOMA-IR were logarithmically transformed before statistical analysis.

***HOMA-B***, homeostasis model of pancreatic beta cell function; ***HOMA-IR***, homeostasis model of insulin resistance; ***A1c***, glycated hemoglobin; ***SE***, standard error; ***CI***, confidence interval; ***P***, P-value.

We then aimed to replicate the significant association of rs6921438 with T2D in another European population. However, the association between SNP rs6921438 and T2D risk was not significant in a T2D case-control study including 3,524 Danish T2D patients and 2,600 Danish controls (OR = 1.02 [0.94;1.10]; *P* = 0.66; **Table 1**). Furthermore, the same SNP did not significantly contribute to variation in fasting glucose, fasting insulin, HOMA-B, HOMA-IR or A1c in 5,621 nondiabetic Danish participants from the Inter99 study (data not shown).

We also did not observe any significant association between the A-allele of the second VEGF-associated SNP rs10738760 (reported to increase circulating VEGF levels [12]) and increased T2D risk (French study: OR = 0.98 [0.91;1.06]; *P* = 0.63; Danish study: OR = 1.04 [0.96;1.12]; *P* = 0.40; **Table 1**). No significant effect of the same allele on variation in fasting glucose, fasting insulin, HOMA-B, HOMA-IR or A1c was observed (*P*>0.05; **Table 2**).

### Effect of SNPs rs6921438 and rs10738760 on T2D-related microvascular complications and variation in related traits

We next assessed the effect of both SNPs on the presence of diabetic nephropathy in two French case-control studies (Corbeil and D2NG), including a total of 1,242 T2D patients with nephropathy and 860 T2D controls. We did not observe any significant association, either in the case-control analyses or in the combined analysis (*P*>0.05; **Table 3**).

**Table 3:** Association of SNPs rs6921438 and rs10738760 with the risk of T2D micro-vascular complications in the Corbeil and Diab2-Néphrogène (D2NG) studies.

SNPs	Studies		*N*	YY (%)	YX (%)	XX (%)	OR (95% CI)*	*P*
**rs6921438** (G-allele = X A-allele = Y)	**Nephropathy**	Controls (Corbeil)	561	126 (22.5)	279 (49.7)	156 (27.8)	-	-
		Cases (Corbeil)	683	139 (20.4)	327 (47.9)	217 (31.8)	1.14 (0.97;1.34)	0.10
		Controls (D2NG)	286	66 (23.1)	129 (45.1)	91 (31.8)	-	-
		Cases (D2NG)	547	108 (19.7)	250 (45.7)	189 (34.6)	1.12 (0.92;1.37)	0.26
		Combined analysis	-	-	-	-	-	0.064
	**Retinopathy**	Controls (Corbeil)	581	117 (20.1)	288 (49.6)	176 (30.3)	-	-
		Cases (Corbeil)	500	110 (22.0)	236 (47.2)	154 (30.8)	0.97 (0.82;1.15)	0.75
		Controls (D2NG)	632	123 (19.4)	318 (50.4)	191 (30.2)	-	-
		Cases (D2NG)	833	174 (20.8)	383 (46.0)	276 (33.2)	1.08 (0.93;1.27)	0.32
		Combined analysis	-	-	-	-	-	0.63
**rs10738760** (A-allele = X G-allele = Y)	**Nephropathy**	Controls (Corbeil)	559	145 (25.9)	269 (48.1)	145 (25.9)	-	-
		Cases (Corbeil)	680	184 (27.1)	321 (47.2)	175 (25.7)	0.96 (0.82;1.12)	0.61
		Controls (D2NG)	284	71 (25.0)	151 (53.2)	62 (21.8)	-	-
		Cases (D2NG)	543	144 (26.5)	249 (45.9)	150 (27.6)	1.07 (0.88;1.31)	0.48
		Combined analysis	-	-	-	-	-	0.97
	**Retinopathy**	Controls (Corbeil)	580	166 (28.6)	262 (45.2)	152 (26.2)	-	-
		Cases (Corbeil)	501	121 (24.1)	255 (50.9)	125 (25.0)	1.06 (0.90;1.25)	0.49
		Controls (D2NG)	629	150 (23.8)	323 (51.4)	156 (24.8)	-	-
		Cases (D2NG)	827	210 (25.4)	407 (49.2)	210 (25.4)	1.02 (0.87;1.20)	0.78
		Combined analysis	-	-	-	-	-	0.51

* OR from additive logistic regression models adjusted for T2D duration and gender.

***OR***, odds ratio; ***CI***, confidence interval; ***P***, P-value.

We also assessed the effect of both SNPs on the presence of diabetic retinopathy in the same French case-control studies, including a total of 1,336 T2D patients with a retinopathy and 1,231 T2D controls. Again, no significant association was observed, in either of the two case-control analyses or in the combined analysis (*P*>0.05; **Table 3**). Furthermore, we did not observe any significant association between rs6921438 or rs10738760 and macular edema in patients from the D2NG study or Corbeil cohort (data not shown). Of note, use of subject age instead of T2D duration in the adjusted regression model did not modify the results for either diabetic retinopathy or diabetic nephropathy (data not shown).

Finally, we assessed the association of both SNPs and the variation in quantitative traits related to T2D microvascular complications (*i.e.* eGFR and ACR) in a total of 3,480 French T2D participants from Corbeil and D2NG. No significant association was observed in these analyses (*P*>0.05; **Table 4**).

**Table 4:** Effect of SNPs rs6921438 and rs10738760 on variation in quantitative traits related to T2D complications in type 2 diabetic participants from the Corbeil and Diab2-Néphrogène (D2NG) studies.

				Mean/median data level by genotype				
SNPs	Quantitative traits	Studies	*N*	YY	YX	XX	β (SE)*	*P*
**rs6921438** (G-allele = X A-allele = Y)	**eGFR (ml/min/1.73 m^2^)**	Corbeil	1,932	76.4±21.2	77.5±20.9	77.9±22.5	0.66 (0.67)	0.32
		D2NG	1,477	74.5±29.8	72.0±27.1	72.9±28.1	−0.89 (0.94)	0.35
		Combined analysis	3,409	-	-	-	-	0.80
	**ACR (mg/mmol)**	Corbeil	1,932	1.7 (0.8;5.0)	1.6 (0.8;5.6)	1.6 (0.8;5.4)	0.023 (0.047)	0.62
		D2NG	1,448	3.1 (0.9;21.3)	2.5 (1.0;16.0)	3.3 (1.0;15.1)	−5.8 (4.5)	0.20
		Combined analysis	3,380	-	-	-	-	0.63
**rs10738760** (A-allele = X G-allele = Y)	**eGFR (ml/min/1.73 m^2^)**	Corbeil	1,929	76.3±22.1	77.2±21.5	78.6±20.3	1.0 (0.7)	0.12
		D2NG	1,468	73.7±30.6	72.0±26.5	73.0±28.1	−0.28 (0.95)	0.77
		Combined analysis	3,397	-	-	-	-	0.28
	**ACR (mg/mmol)**	Corbeil	1,929	1.6 (0.8;5.5)	1.7 (0.8;5.4)	1.7 (0.8;5.0)	−0.018 (0.046)	0.70
		D2NG	1,438	3.5 (1.1;21.6)	2.6 (0.9;14.1)	2.8 (1.0;19.1)	−3.5 (4.7)	0.45
		Combined analysis	3,367	-	-	-	-	0.69

* Per X-allele effect size: coefficient β from additive linear regression models adjusted for T2D duration and gender.

Data are presented as mean ± standard deviation or median (interquartile range). Data for ACR were logarithmically transformed before statistical analysis.

***eGFR***, estimated glomerular filtration rate using modification of diet in renal disease (MDRD) formula; ***ACR***, urinary albumin/creatinine ratio; ***T2D***, type 2 diabetes; ***SE***, standard error; ***CI***, confidence interval; ***P***, P-value.

## Discussion

We initially observed a strong association between the G-allele of rs6921438 (the allele increasing VEGF levels in the general population [12]), and increased T2D risk (BMI-adjusted or not) in the French population. This result was in line with some studies which reported that T2D patients show higher VEGF levels compared with normoglycemic individuals [12], [24], [25]. Furthermore, we found the same allele to be associated with increased A1c levels in a French general population sample.

However, we did not confirm these findings in the Danish population. Of note, the frequency of the G-allele was higher in the French population than in the Danes (52.5% *versus* 50.8% in controls/55.6% *versus* 51.8% in cases). Therefore, there may be a geographic effect on the frequency of this SNP. Nevertheless, in the international DIAGRAM consortium which performed meta-analysis of GWAS for T2D risk, the SNP rs6921438 was not significantly associated with T2D (*N_cases_* = 8,130; *N_controls_* = 38,987) [26]. Furthermore, no significant effect for rs6921438 on A1c (*N* = 46,368) [27] or fasting glucose levels (*N* = 46,186) [28] was observed by in the analyses conducted by the MAGIC consortium which performed meta-analysis of GWAS for glucose- and insulin-related traits. Thus, the significant association in the French study is likely to be a false positive result. The second VEGF-associated SNP rs10738760 was not associated with T2D (or related metabolic traits) either in the French individuals or in the Danes.

Furthermore, we did not find any significant association of VEGF-related SNPs rs6921438 or rs10738760 on the presence of diabetic microvascular complications or variation in related traits. These results are in line with the data from the CKDGen and CARe Renal consortia which did not show any significant contribution of these SNPs to eGFR, ACR or microalbuminuria [29], [30], [31]. Of note, the CDKGen consortium identified a genome-wide significant effect of SNP rs881858 located close to *VEGFA* (and SNP rs6921438) on eGFR variation [31]. However, SNPs rs881858 and rs6921438 were not in linkage disequilibrium in the HapMap European (CEU) population (r^2^ = 0.0/D′ = 0.1).

Therefore, in the present study, we were unable to find a direct link between SNPs rs6921438 and rs10738760, which explain almost half of the variance in circulating VEGF [12], and risk of T2D, diabetic nephropathy or more importantly, diabetic retinopathy including macular edema. A limitation of this study which should be taken into consideration would be a lack of statistical power, to some extent (**Table 5**). However, even if the size of the present study (continuous or case-control) was relevant, we were not able to identify even marginally significant effects (**Table 5**).

**Table 5:** Case numbers needed for reaching a statistical power of 80% according to the expected odds ratio or effect.

Study	Statistical Power	|β|	OR	*N_min_* [*N_study_*]
**Fasting glucose** *(continuous)*	80%	0.03 mmol/l	NA	4,364 [4,760]
**Fasting insulin** *(continuous)*	80%	0.03 pmol/l	NA	4,364 [4,760]
**Nephropathy** *(case-control)*	80%	NA	1.18	1,157 [1,242]
**Retinopathy** *(case-control)*	80%	NA	1.17	1,285 [1,336]
**eGFR** *(continuous)*	80%	1,42 ml/min/1.73 m^2^	NA	3,567 [3,480]
**ACR** *(continuous)*	80%	0.10 mg/mmol	NA	3,534 [3,480]

Only statistical power of association analyses with a P-value above 0.05 was analysed.

***β***, effect size; ***OR***, odds ratio; ***NA***, not applicable; ***eGFR***, estimated glomerular filtration rate using modification of diet in renal disease (MDRD) formula; ***ACR***, urinary albumin/creatinine ratio.

Of note, the effect of VEGF was previously clearly demonstrated in diabetic macular edema [32]. Thus, our current negative result must be considered with caution. Factors driving VEGF locally may be different from those significantly associated with variation in plasma levels. Ultimately, VEGF might also be regulated in the retina by factors largely exceeding the impact of genetic determinants.

If confirmed in other studies with VEGF levels and clinical phenotypes related to diabetic complications, these findings would show that the link between VEGF and T2D and its complications might be indirect and more complex than expected.

## Supporting Information

Table S1 **Clinical characteristics of the population studies.** Data are presented as mean ± standard deviation or median (interquartile range). ***NA***, not applicable or not available; ***BMI***, body mass index; ***HbA1c***, glycated hemoglobin; ***eGFR***, estimated glomerular filtration rate using modification of diet in renal disease (MDRD) formula; ***ACR***, urinary albumin/creatinine ratio; ***T2D***, type 2 diabetes; ***D2NG***, Diab2-Néphrogène; ***retino***, case-control study for retinopathy risk; ***nephro***, case-control study for nephropathy risk.
(DOCX)
