# Supplementary material for: What Is the Contribution of Two Genetic Variants Regulating VEGF Levels to Type 2 Diabetes Risk and to Microvascular Complications?
Source: PLoS One. 2013 Feb 6;8(2):e55921. doi: 10.1371/journal.pone.0055921 (PMC3566098; doi:10.1371/journal.pone.0055921)
Supplement: Table S1 — Clinical characteristics of the population studies. Data are presented as mean ± standard deviation or median (interquartile range). NA, not applicable or not available; BMI, body mass index; HbA1c, glycated hemoglobin; eGFR, estimated glomerular filtration rate using modification of diet in renal disease (MDRD) formula; ACR, urinary albumin/creatinine ratio; T2D, type 2 diabetes; D2NG, Diab2-Néphrogène; retino, case-control study for retinopathy risk; nephro, case-control study for nephropathy risk. (DOCX) [file pone.0055921.s001.docx]

**Table S1. Clinical characteristics of the population studies**

| **Studies** | **D.E.S.I.R.**  *(nondiabetic)* | **Corbeil**  *(T2D)* | | **D2NG**  *(T2D)* | | **French T2D case-control study** | |  | **Danish T2D case-control study** | |
| --- | --- | --- | --- | --- | --- | --- | --- | --- | --- | --- |
|  |  |  |  |  |  | **T2D cases** | **Controls** |  | **T2D cases** | **Controls** |
| *N* (M/F) | 4,760  (2,330 / 2,430) | 1,970  (1,189 / 780) | | 1,510  (917 / 593) | | 7,020  (4,619 / 2,401) | 3,875  (1,774 / 2,101) |  | 3,561  (2,120 / 1,441) | 2,623  (1,222 / 1,401) |
| Age (years) | 47.2 ± 10.0 | 60.4 ± 10.5 | | 64.3 ± 10.0 | | 62.9 ± 10.1 | 53.7 ± 5.6 |  | 60.4 ± 9.7 | 53.1 ± 5.8 |
| BMI (kg/m²) | 24.6 ± 3.7 | 31.1 ± 5.6 | | 31.0 ± 5.7 | | 30.0 ± 5.3 | 24.7 ± 3.5 |  | 30.7 ± 5.5 | 25.8 ± 4.0 |
| FG (mmol/l) | 5.2 ± 0.5 | NA | | NA | | NA | NA |  | NA | NA |
| Fasting insulin (pmol/l) | 39.2 (28.5;55.9) | NA | | NA | | NA | NA |  | NA | NA |
| HbA1c (%) | 5.43 ± 0.40 | NA | | NA | | NA | NA |  | NA | NA |
| eGFR (ml/min/1.73m²) | NA | 77.3 ± 21.4 | | 72.7 ± 28.0 | | NA | NA |  | NA | NA |
| ACR (mg/mmol) | NA | 1.7 (0.8;5.3) | | 2.9 (1.0;16.7) | | NA | NA |  | NA | NA |
| T2D duration (years) | NA | 11.7 ± 8.9 | | 15.4 ± 10.2 | | NA | NA |  | NA | NA |
| eGFR retino  Controls *vs* cases | NA | 76.2 ± 20.7 | 71.7 ± 25.3 | 79.2 ± 25.9 | 68.2 ± 28.7 | NA | NA |  | NA | NA |
| ACR retino  Controls *vs* cases | NA | 1.7 (2.5) | 4.0 (16.1) | 1.5 (5.4) | 6.0 (34.6) | NA | NA |  | NA | NA |
| T2D duration retino  Controls *vs* cases | NA | 17.3 ± 7.0 | 16.7 ± 8.9 | 18.3 ± 9.5 | 18.7 ± 9.2 | NA | NA |  | NA | NA |
| eGFR nephro  Controls *vs* cases | NA | 79.0 ± 19.6 | 71.3 ± 24.1 | 81.3 ± 21.9 | 62.0 ± 30.1 | NA | NA |  | NA | NA |
| ACR nephro  Controls *vs* cases | NA | 1.0 (1.0) | 9.6 (24.1) | 1.1 (1.2) | 17.4 (75.2) | NA | NA |  | NA | NA |
| T2D duration nephro  Controls *vs* cases | NA | 17.6 ± 7.0 | 15.1 ± 9.3 | 18.3 ± 9.7 | 19.1 ± 9.1 | NA | NA |  | NA | NA |

Data are presented as mean ± standard deviation or median (interquartile range).

***NA***, not applicable or not available; ***BMI***, body mass index; ***HbA1c***, glycated hemoglobin; ***eGFR***, estimated glomerular filtration rate using modification of diet in renal disease (MDRD) formula; ***ACR***, urinary albumin/creatinine ratio; ***T2D***, type 2 diabetes; ***D2NG***, Diab2-Néphrogène; ***retino***, case-control study for retinopathy risk; ***nephro***, case-control study for nephropathy risk.
